# Deep learning for scalable atomic electron tomography

**DOI:** 10.1093/nsr/nwag561

**Published:** 2026-08-29

**Authors:** Jianwei Miao

**Affiliations:** Department of Physics and Astronomy and California NanoSystems Institute, University of California, USA

Determining the three-dimensional (3D) arrangement of atoms is fundamental to understanding the structure and properties of matter. Established methods such as crystallography and single-particle cryo-electron microscopy rely on crystalline order or averaging over many identical structures. Atomic electron tomography (AET) addresses these limitations by determining the 3D coordinates and elemental identities of individual atoms in a single nanoscale object from a series of electron micrographs recorded as the specimen is tilted [1,2]. AET can directly reveal defects, interfaces, grain boundaries and amorphous atomic arrangements. It has enabled quantitative studies of dislocations and strain, chemical order and disorder in complex alloys, crystal nucleation, active sites in nanocatalysts and atomic packing in amorphous materials [1,2]. By linking local atomic structure to electronic, mechanical and catalytic properties, AET provides new insights into how materials form, function and fail. Its broader use remains limited by incomplete angular sampling, electron-beam damage and the narrow depth of field at atomic resolution. Missing angles leave gaps in the measured information, while the limited depth of field prevents atoms throughout a thick specimen from being sharply imaged at the same time.

Deep learning can reduce the effects of incomplete tomographic measurements, but supervised methods usually require large paired datasets in which incomplete or degraded reconstructions are matched to known reference images or volumes [3,4]. In AET, experimental 3D ground truth is generally unavailable because the true atomic coordinates of a specimen are unknown. Training data are therefore often generated from simulated atomic models. For large and structurally complex materials, such simulations are computationally demanding and depend on assumptions about the atomic structure and imaging physics. In a recent *National Science Review* paper, Li *et al*. address this limitation with a data-driven framework that uses abundant 2D electron micrographs to generate paired training data [5]. Each micrograph is treated as a pseudo-3D object one voxel thick along the tilt axis and numerically rotated to generate simulated projections. The framework incorporates incomplete angular sampling and, for thick-specimen AET, depth-dependent defocus. The projections are first reconstructed with an iterative algorithm, and a standard 2D U-Net is trained to map the degraded reconstruction toward the original object (Fig. 1). The key idea is that electron micrographs provide broad statistical diversity, while retaining structural features found in real microscopic images. The authors’ analyses are consistent with reduced dataset-specific memorization, while tests across different materials, image sizes and spatial resolutions demonstrate transfer within the range examined in the study.

**Figure 1.**
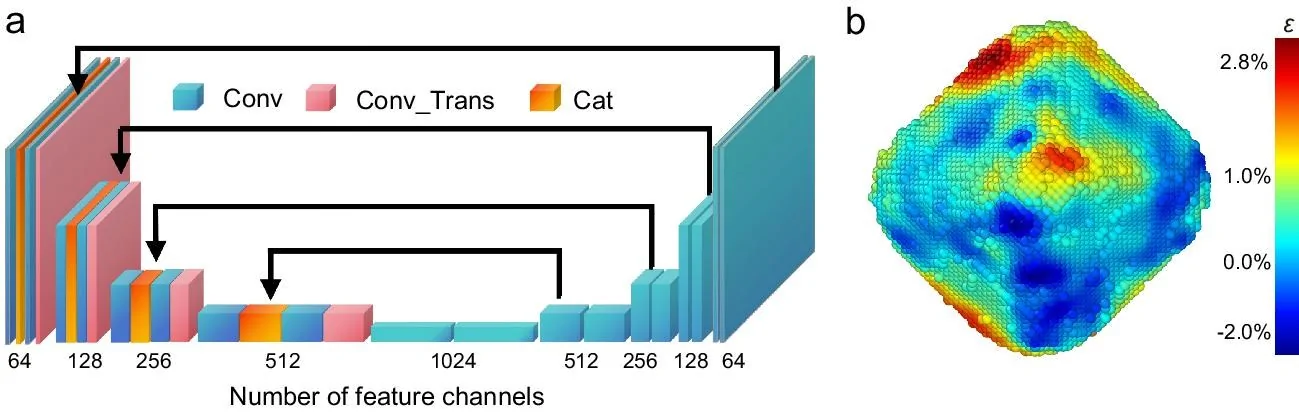
Deep-learning architecture and 3D atomic reconstruction of a 13-nm-thick Pt nanoparticle. (a) 2D U-Net used to improve the initial tomographic reconstruction. The encoder extracts features at multiple scales, while the decoder restores spatial detail through transposed convolutions and skip connections. Numbers denote feature channels. Conv, convolution; Conv_Trans, transposed convolution; Cat, concatenation. (b) 3D atomic model of a 13-nm octahedral Pt nanoparticle reconstructed from 48 experimental projections, containing 52 138 atoms. Atoms are colored by local lattice strain, ε, relative to bulk Pt, with blue and red indicating compression and expansion, respectively. Split-data cross-validation gives a 3D coordinate RMSD of 23 pm and 98.2% matched atoms. Adapted with permission from Ref. [5].

The experimental demonstration of this framework is AET of a 13-nm octahedral platinum nanoparticle. At this thickness, only part of the particle can be sharply focused at a time. Using 48 experimental projections, the authors identify 52 138 atoms. Split-data cross-validation estimates a 3D coordinate root-mean-square deviation (RMSD) of 23 pm, with 98.2% of the atoms matched between two independent reconstructions. The same analysis using the real-space iterative reconstruction algorithm [6] gives an RMSD of 29 pm and a common-atom ratio of 96.3%. The improvement is especially important at the poorly focused surface, where the method enables more reliable determination of the atomic structure needed for structure–property correlations. These results show that incorporating depth-dependent imaging physics into deep learning can improve 3D atomic reconstruction of relatively thick specimens. This study provides an important step toward scalable, data-driven AET by reducing the need for material-specific 3D training models and explicitly accounting for incomplete angular sampling and depth-dependent defocus. Reliable atomic coordinates, however, still depend on carefully performed experiments and a rigorous, integrated workflow in which image preprocessing, projection alignment, tomographic reconstruction, atom tracing and quantitative validation collectively contribute to the accuracy of the final 3D atomic model [7]. Complementary work by the same group combines a generative atomic prior with iterative enforcement of the measured projections, enabling AET from sparse and limited-angle data across the tested materials without retraining [8].

Looking ahead, ptychographic AET (pAET) is likely to play a central role in the future of the field [9]. Ptychography replaces conventional lens-based image formation with coherent diffraction measurements and computational phase retrieval, providing high dose efficiency and strong sensitivity to light elements [1]. Multi-slice ptychography further models electron propagation through successive layers of a specimen, enabling pAET to image samples substantially thicker than the conventional probe depth of field [10]. Combining deep learning with multi-slice pAET will further extend 3D atomic imaging to larger, lighter and more beam-sensitive materials.
